# Engineered exosomes for diabetic foot ulcers: lessons learned, challenges remain^☆^

**DOI:** 10.1016/j.mmr.2026.100022

**Published:** 2026-04-15

**Authors:** Hyun-Do Jung

**Affiliations:** Division of Materials Science and Engineering, Hanyang University, Seoul 04763, Republic of Korea

**Keywords:** Exosomes, Trace element, Diabetic foot ulcers, Stimuli-responsive hydrogel

Diabetic foot ulcers (DFUs) remain trapped in a self-reinforcing loop of inflammation, oxidative stress, and impaired perfusion, and recurrence is common despite debridement, offloading, antibiotics, and conventional dressings. Wang *et al*. [1] frame DFU as an “inflammation-ischemia” vicious cycle and argue that symptom-centric strategies often fail to reset the wound microenvironment. This challenge is amplified in military and disaster situations, where wound care must be delivered with limited infrastructure, constrained follow-up, and heightened contamination risk. Prolonged Field Care guidance emphasizes practical wound protection, infection control, and approaches that remain functional when resources are austere [2]. These realities have accelerated interest in therapies that are i) field-deployable, ii) manufacturable at scale, and iii) robust against hostile wound milieus.

Within regenerative medicine, a major recent trend is the shift from fragile living-cell therapies toward cell-free biologics, particularly extracellular vesicles/exosomes (Exo), due to their paracrine potency and potential for standardized production [3]. However, there are still challenges, including scalable, reproducible yield and effective local delivery/retention in protease- and reactive oxygen species (ROS)-rich chronic wounds [4]. This is exactly where Wang *et al*. [1] position their contribution, an integrated strategy combining trace element (TE) programming, three-dimensional (3D) dynamic culture, and a stimuli-responsive hydrogel to address both potency and delivery constraints.

By combining trace-element programming with 3D dynamic culture, the authors establish a scalable approach to engineering exosomes from human umbilical cord-derived mesenchymal stem cells. This strategy yields a marked increase in exosome production, addressing one of the most persistent translational bottlenecks in the field-manufacturing efficiency, while remaining conceptually aligned with the broader shift toward 3D culture and bioreactor-like systems. Importantly, positioning this advance within the framework of emerging community standards such as MISEV2023 underscores its relevance not merely as a yield optimization, but as a step toward more standardized and comparable extracellular vesicle production [5].

Rather than reiterating generalized antibacterial effects, the authors focus on immune microenvironment modulation, supported by integrated proteomic and transcriptomic analyses. Enrichment of complement 1q binding protein (C1QBP) in engineered exosomes links trace-element programming to complement-associated pathways, and functional experiments converge on a complement-mitochondria-autophagy axis that mitigates oxidative stress. The attenuation of pro-proliferative effects following *C1QBP* knockdown strengthens causal interpretation, moving the field beyond phenomenological descriptions toward mechanistic clarity.

The OHA-LACS-ultra vilollet (UV) hydrogel loaded with 3D-TE-Exo (OLUE) hydrogel platform couples dynamic Schiff-base chemistry with UV-responsive disulfide networks to enable stimulus-adaptive release of exosomes under high-glucose and high-ROS conditions characteristic of diabetic foot ulcers. This environment-responsive behavior, together with robust *in vivo* wound closure, highlights how rational material design can amplify the therapeutic relevance of engineered exosomes. Such dressing-like systems are particularly compelling for constrained-care settings, where early modulation of wound trajectories can yield disproportionate clinical benefit.

Despite these advances in manufacturing, mechanism, and delivery, several translational challenges remain that must be addressed before engineered exosome systems can be considered field-ready therapies. First, the reported 29-fold increase in particle yield is encouraging, but translation will ultimately hinge on potency-defined critical quality attributes (CQAs) rather than particle counts alone. Establishing DFU-relevant release criteria, such as migration/angiogenesis assays under high-glucose/ROS conditions, together with a minimal cargo signature linked to mitochondrial rescue and immunomodulation, would strengthen comparability across donors and manufacturing scales, in line with the direction advocated by MISEV2023. Second, multi-trace-element programming is conceptually attractive yet expands the variable space for reproducibility and safety. A design-of-experiments approach to identify a minimal TE set and concentration window, coupled with specification limits for TE-related exosomal signatures and residual elemental content, would reduce batch-to-batch uncertainty. Third, immunomodulation targeting complement signaling may be context-dependent. DFUs are frequently colonized or infected, and excessive dampening of innate pathways could compromise pathogen clearance. Future studies should therefore evaluate wound closure and microbial burden in infected/biofilm DFU models and consider rational combination strategies when antimicrobial support is required. Fourth, the “initiator-free UV” dual-network hydrogel is elegant, but field deployability depends on the practical curing scenario. Preformed sterile patches, visible-light/self-curing alternatives, and room-temperature stability should be prioritized to align with austere-care constraints. Finally, bridging from streptozocin rodents to clinically faithful DFU will require staged validation in ischemic/neuropathy models and large animals, with endpoints beyond closure. Consequently, addressing these manufacturing, mechanistic, and deployment bottlenecks will determine whether engineered exosome-hydrogel systems can evolve into genuinely field-ready regenerative products.

The next phase of clinical translation, particularly in resource-constrained or austere-care settings, will likely depend on: 1) standardized CQAs and scale-up workflows, 2) validation in ischemic/infected, clinically faithful models, and 3) ruggedized, low-complexity formulation/delivery compatible with austere wound care pathways.

## Abbreviations

3D: Three-dimensional

DFU: Diabetic foot ulcers

Exo: Exosome

ROS: Reactive oxygen species

TE: Tissue engineering

## Ethics approval and consent to participate

Not applicable.

## Author contributions

HDJ solely contributed to the conception, design, analysis, and drafting of the manuscript. The author read and approved the final manuscript.

## Funding

This work was supported by the National Research Foundation of Kore grant funded by the Korea government (RS-2024-00405381; RS-2025-00513935), the Korea Institute of Marine Science and Technology Promotion funded by the Ministry of Oceans and Fisheries (RS-2024-00405273); and the Korean Fund for Regenerative Medicine (the Ministry of Science and ICT, the Ministry of Health and Welfare, KFRM 24A0105L1).

## Competing interest

The author declares that there is no conflict of interest.

## Data Availability

Not applicable.
